# Glucagon-like peptide-1 receptor signaling deficiency exacerbates hematopoietic stem cell graft rejection in mice

**DOI:** 10.1093/jimmun/vkaf251

**Published:** 2025-09-24

**Authors:** Mark Rusznak, Daniela Sierra-Hernandez, Catherine Dupuy, Shinji Toki, Ashley Y Wu, Uttam Rao, Masako Abney, Jian Zhang, Qianni Hu, Christian M Warren, Daniel J Drucker, Kevin D Niswender, Brian Engelhardt, Tae Kon Kim, Katherine N Gibson-Corley, Katherine N Cahill, Kenneth R Cooke, R Stokes Peebles

**Affiliations:** Division of Allergy, Pulmonary, and Critical Care Medicine, Department of Medicine, Vanderbilt University Medical Center, Nashville, TN, United States; Department of Pathology, Microbiology, and Immunology, Vanderbilt University Medical Center, Nashville, TN, United States; Division of Allergy, Pulmonary, and Critical Care Medicine, Department of Medicine, Vanderbilt University Medical Center, Nashville, TN, United States; Division of Allergy, Pulmonary, and Critical Care Medicine, Department of Medicine, Vanderbilt University Medical Center, Nashville, TN, United States; Division of Allergy, Pulmonary, and Critical Care Medicine, Department of Medicine, Vanderbilt University Medical Center, Nashville, TN, United States; Division of Allergy, Pulmonary, and Critical Care Medicine, Department of Medicine, Vanderbilt University Medical Center, Nashville, TN, United States; Division of Hematology Oncology, Department of Medicine, Vanderbilt University Medical Center, Nashville, TN, United States; Division of Allergy, Pulmonary, and Critical Care Medicine, Department of Medicine, Vanderbilt University Medical Center, Nashville, TN, United States; Division of Allergy, Pulmonary, and Critical Care Medicine, Department of Medicine, Vanderbilt University Medical Center, Nashville, TN, United States; Division of Hematology Oncology, Department of Medicine, Vanderbilt University Medical Center, Nashville, TN, United States; United States Department of Veterans Affairs, Tennessee Valley Healthcare System, Nashville, TN, United States; Lunenfeld-Tanenbaum Research Institute, Mount Sinai Hospital, University of Toronto, Toronto, ON, Canada; Division of Diabetes, Endocrinology, and Metabolism, Department of Medicine, Vanderbilt University Medical Center, Nashville, TN, United States; Division of Hematology Oncology, Department of Medicine, Vanderbilt University Medical Center, Nashville, TN, United States; Department of Pathology, Microbiology, and Immunology, Vanderbilt University Medical Center, Nashville, TN, United States; Division of Hematology Oncology, Department of Medicine, Vanderbilt University Medical Center, Nashville, TN, United States; Department of Pathology, Microbiology, and Immunology, Vanderbilt University Medical Center, Nashville, TN, United States; Division of Allergy, Pulmonary, and Critical Care Medicine, Department of Medicine, Vanderbilt University Medical Center, Nashville, TN, United States; Department of Oncology, Sidney Kimmel Cancer Center, Johns Hopkins University, School of Medicine, Baltimore, MD, United States; Division of Allergy, Pulmonary, and Critical Care Medicine, Department of Medicine, Vanderbilt University Medical Center, Nashville, TN, United States; Department of Pathology, Microbiology, and Immunology, Vanderbilt University Medical Center, Nashville, TN, United States; United States Department of Veterans Affairs, Tennessee Valley Healthcare System, Nashville, TN, United States

**Keywords:** HSCT, GF, GLP1R, rejection

## Abstract

Graft failure (GF) following hematopoietic stem cell transplantation (HSCT) remains a major complication particularly in the setting of human leukocyte antigen (HLA)-mismatched grafts where residual host lymphocytes can drive immune-mediated rejection. While strategies to mitigate GF have been explored, such as intensified conditioning or donor T cell supplementation, these approaches carry significant risks, including increased toxicity and graft-versus-host disease (GVHD). Recent studies have highlighted the glucagon-like peptide-1 receptor (GLP1R) as a critical regulator of immune homeostasis, yet its role in HSC engraftment remains unexplored. Here, we demonstrated that GLP1R deficiency in recipient mice leads to a profound increase in GF following MHC-mismatched allogeneic HSCT. Although GLP1R knockout (GLP1RKO) and wild-type (WT) mice exhibited comparable survival and engraftment following syngeneic or minor antigen-mismatched transplants, GLP1RKO mice undergoing MHC-mismatched HSCT experienced significantly greater weight loss, earlier mortality, and reduced donor chimerism. Histologic and cytokine analyses confirmed that this phenotype is not driven by GVHD, but rather by early graft rejection. Depletion of CD90^+^ recipient T cells prior to transplantation rescued engraftment in GLP1RKO mice, further supporting a model in which GLP1R signaling restrains host lymphocyte-mediated graft rejection. These findings identify GLP1R as a novel regulator of allogeneic HSC engraftment and suggest that GLP1R agonists, widely used for metabolic disorders, may have therapeutic potential in preventing HSC graft rejection. Given the lack of targeted interventions for HSC graft rejection, further studies are warranted to investigate GLP1R-directed therapies in the context of allogeneic HSCT.

## Introduction

Graft failure (GF) following hematopoietic stem cell transplantation (HSCT) is a serious complication that occurs in approximately 3.8% to 5.6% of HSCT recipients.^1–3^ GF is an inability of donor HSCs to repopulate the hematopoietic compartment in a conditioned recipient and is defined by a patient’s absolute neutrophil count (ANC) never exceeding 0.5 × 10^9^/l for at least 3 consecutive days.^4^ Allogeneic HSCT is a curative option for a variety of malignant and non-malignant disorders, and there are many variable factors in the procedure. As such, certain conditions and treatment approaches confer increased risk for GF in the setting of HSCT. For example, transplant for non-malignant disorders, human leukocyte antigen (HLA)-mismatched grafts, reduced intensity conditioning regimens, female grafts for male recipients, and low stem cell dose all increase the risk for GF.^4–6^ The primary immunologic mechanism by which GF occurs is cellular rejection of the HSC graft by residual T cells and natural killer (NK) cells in the recipient.^7^^,^^8^ Host lymphocytes are capable of rejecting both HLA-matched and mismatched HSC grafts, although rejection is more likely with mismatch.^9^^,^^10^

Modifications to the conditioning regimen and graft may reduce the likelihood of GF; increasing the intensity of the conditioning regimen to eliminate the residual host T cells and NK cells is effective in reducing GF.^6^ However, this can have deleterious side effects which can be difficult to justify particularly in the setting of HSCT for non-malignant etiologies. Alternatively, supplementing the allogeneic HSC graft with donor T cells can protect against GF. In this situation, donor T cells can attack residual lymphocytes in the host that may reject the allogeneic HSC graft.^11^^,^^12^ The obvious shortcoming of this approach is the increased risk of graft-versus-host disease (GVHD), especially in the case of non-malignant diseases where a graft-versus-leukemia (GVL) effect is not relevant.^13^^,^^14^ Several genes and their protein products have been implicated in the pathophysiology of GF following allogeneic HSCT. The most well-known include the HLA genes and killer cell immunoglobulin-like receptors (KIRs).^15^^,^^16^ Others like interferon gamma (IFN-γ) have been associated with increased cellular rejection of HSC grafts,^17^ and stem cell boosts with CD34^+^ HSCs have been used as treatments for GF.^18^ Despite advances in understanding the pathophysiology of GF, targeted therapies that prevent GF without impeding lymphocyte recovery are lacking.^19^ For this reason, it is critical to identify new genes relevant in the pathophysiology of GF that might serve as therapeutic targets.

In recent years, the glucagon-like peptide-1 receptor (GLP1R) has garnered significant interest, largely due to the success of GLP1R agonists in managing type 2 diabetes and promoting weight loss.^20–23^ Beyond their well-documented metabolic benefits, GLP1R signaling has been increasingly recognized for its role in modulating various forms of pathological inflammation,^24^ including gastrointestinal inflammation,^25–27^ allergic inflammation,^28–30^ and neuroinflammatory conditions.^31–33^ The extensive anti-inflammatory properties associated with GLP1R signaling have fueled growing interest in exploring the therapeutic potential of GLP1R agonists for inflammatory diseases, as reflected in numerous ongoing clinical trials.^34–37^ A recent report described the importance of GLP1R signaling in the survival of solid organ allografts.^38^ It concluded that GLP1R signaling deficiency in mice resulted in accelerated T cell-mediated rejection of heart allografts compared to WT controls. Inspired by these results, we hypothesized that GLP1R signaling deficiency may similarly exacerbate allogeneic HSC graft rejection.

## Materials and methods

### Mice

All in vivo and in vitro mouse experiments utilized age-matched, female mice that are 8 to 16 wk old. Mice were housed in a temperature-controlled room at 22.2 °C on a half day light-dark cycle. Mice were fed a regular diet (PicoLab^®^ Laboratory Rodent Diet 5LOD), and tap water was available ad libitum. Mouse experiments were approved by the Institutional Animal Care and Use Committee (IACUC) at Vanderbilt University Medical Center and were conducted according to the guidelines for the Care and Use of Laboratory Animals prepared by the Institute of Laboratory Animal Resources, National Research Council. BALB/c and C3.SW-H2^b^/SnJ mice were purchased from Jackson Laboratories and housed in our colony until the time of experimentation. GLP1RKO and WT controls on the C57BL/6J background were bred and maintained in our colony. The creation and characterization of these mice has been previously described.^39^^,^^40^

### Isolation of bone marrow-derived HSCs

Bone marrow-derived HSCs were harvested from the tibias and femurs of donor mice (C57BL/6J WT, BALB/c WT, C3.SW-H2^b^/SnJ). Briefly, bones were dissected from mice euthanized with pentobarbital overdose. Bones were cleaned of all soft tissue and cut at the ends with a scalpel. Up to 4 bones were placed in 0.5 ml snap tubes that had been punctured at the bottom with a 19-gauge needle. In brief, 0.5 ml tubes containing bones were closed and placed inside of larger 1.5 ml snap tubes. The combined tubes were centrifuged to collect bone marrow cells. Cells were passed through a 70 μM strainer and erythrocyte lysis was carried out with an ammonium chloride-based lysing reagent (Tonbo^®^). Cell counts were obtained with a hemocytometer and an aliquot of cells diluted in Trypan blue. Bone marrow cells collected from mice of identical strains were pooled to create the HSC graft. All steps after RBC lysis were performed in BMT Media (1640 RPMI w/L-glutamine, RMPI 1640 with 10% fetal bovine serum (FBS), Penicillin/Streptomycin, HEPES, Sodium Pyruvate).

### HSC T cell depletion

HSCs were isolated and depleted of RBCs as described above. Cells were incubated with Mouse Fc Block (BD™), and subsequently with anti-mouse Thy1.2 (CD90.2) (30H12) in BMT Media (5 μg/mL) for 30 min. Lysis of labeled cells was carried out with Low-Tox^®^ -M Rabbit Complement (Cedarlane^®^) diluted 1:120 in BMT Media at 37 °C for 45 min.

### Splenic T cell isolation

Spleens were harvested from donor mice (C57BL/6J WT, BALB/c WT, C3.SW-H2^b^/SnJ) euthanized with pentobarbital overdose. Spleens were smashed through a 70 μM strainer and erythrocyte lysis was carried out with an ammonium chloride-based lysing reagent (Tonbo^®^). Cell counts were obtained with a hemocytometer and an aliquot of cells diluted in Trypan blue. T cells were isolated from splenocytes with the Pan T Cell Isolation Kit II, Mouse (Miltenyi™) according to the manufacturer protocol.

### Bone marrow-derived HSC and splenic T cell transplantation

**Figure 1.**
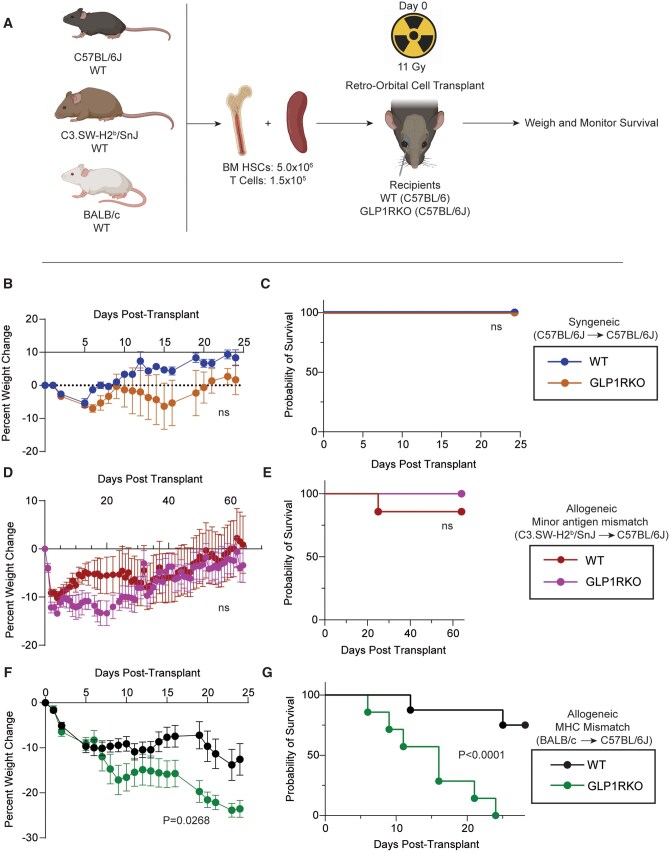
GLP1R deficiency in recipients causes increased weight loss and mortality in MHC-mismatched HSCT. (A) Diagram of transplant model used in Fig. 1. Bone marrow-derived HSCs and splenic T cells were isolated from C57BL/6J (syngeneic), C3.SW-H2^b^/SnJ (allogeneic, minor antigen mismatch), and BALB/c (allogeneic, HSC mismatch) and transplanted into WT or GLP1RKO recipients (C57BL/6J background). Recipients were conditioned with 11 Gy lethal irradiation administered in 2 × 5.5 Gy doses 3 h apart. Also, 5.0 × 10^6^ bone marrow-derived hematopoietic stem cells (BM HSCs) were transferred per graft. And 1.5 × 10^5^ splenic T cells were used in T cell grafts. Transplantation occurred 2 h after the last irradiation. (B) Percent weight change of WT syngeneic and GLP1RKO syngeneic mice. Percent change is calculated relative to the pre-transplant day 0 weight. Mice that die during the measurement period have their final weight included for subsequent days. Error bars indicate standard error of the mean (SEM). Significance was assessed with a 2-way repeated measures ANOVA. ns = no significant difference (*n* = 3 per group). Figure is representative of 2 independent experiments. (C) Kaplan–Meier survival curve of WT syngeneic transplanted and GLP1RKO syngeneic transplanted mice. Significance was assessed with Mantel-Cox (Logrank) test. ns = no significant difference (*n* = 3 per group). Figure is representative of two independent experiments. (D) Percent weight change of WT and GLP1RKO allogeneic (minor antigen mismatch) transplanted mice. Percent change is calculated relative to the pre-transplant day 0 weight. Mice that die during the measurement period have their final weight included for subsequent days. Error bars indicate standard error of the mean (SEM). Significance was assessed with a two-way repeated measures ANOVA. ns = no significant difference (*n*= 7 per group). Figure is representative of 1 experiment. (E) Kaplan–Meier survival curve of WT and GLP1RKO allogeneic (minor antigen mismatch). Significance was assessed with Mantel–Cox (Logrank) test between WT allogeneic and GLP1RKO allogeneic groups. ns = no significant difference (*n* = 7 per group). Figure is representative of 1 experiment. (F) Percent weight change of WT and GLP1RKO allogeneic (MHC mismatch) transplanted mice. Percent change is calculated relative to the pre-transplant day 0 weight. Mice that die during the measurement period have their final weight included for subsequent days. Error bars indicate standard error of the mean (SEM). Significance was assessed with a 2-way repeated measures ANOVA (*n* = 9 WT, *n* = 8 GLP1RKO). Figure is representative of 2 experiments. (G) Kaplan–Meier survival curve of WT and GLP1RKO allogeneic (MHC mismatch). Significance was assessed with Mantel–Cox (Logrank) test between WT allogeneic and GLP1RKO allogeneic groups (*n* = 9 WT, *n* = 8 GLP1RKO). Figure is representative of 2 experiments. Created in https://BioRender.com.

**Figure 2.**
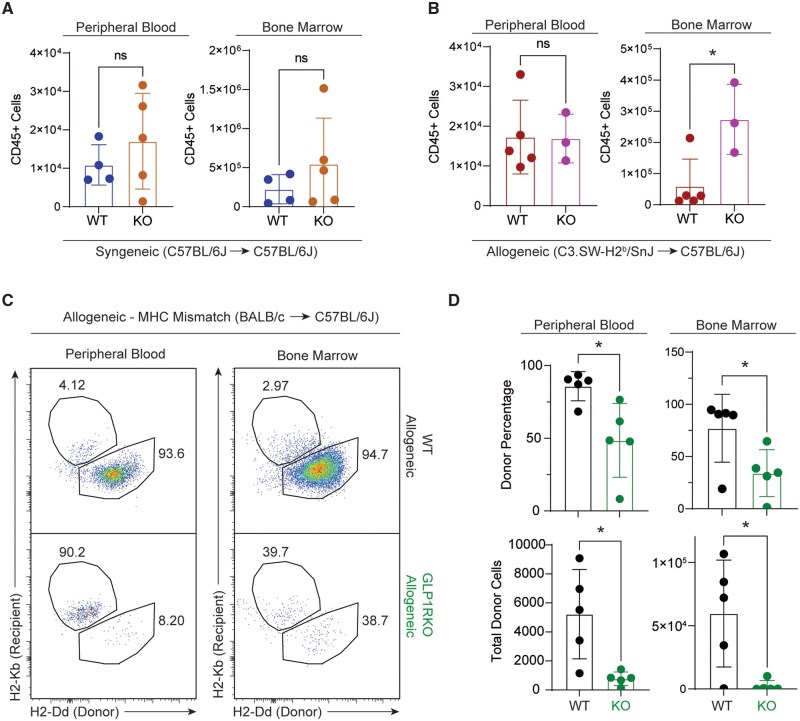
GLP1R deficiency in recipients causes engraftment failure of MHC-mismatched HSCs. (A) Total CD45^+^ cells in the bone marrow and peripheral blood of WT (*n* = 4) and GLP1RKO (*n* = 4) syngeneic transplanted mice at Day 7. Significance was measured with an unpaired Student *t* test. (B) Total CD45^+^ cells in the bone marrow and peripheral blood of WT (*n* = 4) and GLP1RKO (*n* = 3) minor antigen mismatch transplanted mice (C3.SW-H2^b^/SnJ [C3.SW] donors) at day 7. Significance was measured with an unpaired Student *t* test. * = *P* < 0.05. (C) Representative flow cytometry plots of peripheral blood and bone marrow of WT and GLP1RKO allogeneic transplant mice at day 7. Cells are pre-gated on live, CD45+ singlet cells. “Donor” denotes the MHC haplotype originating from BALB/c, while “Recipient” denotes C57BL/6J. (D) Quantification of donor chimerism and total donor cells (BALB/c MHC+) in the peripheral blood and bone marrow of WT (*n* = 5) and GLP1RKO (*n* = 6) allogeneic mice at day 7. Donor percentage is calculated as of ([BALB/c MHC+ CD45+ cells]/[Total CD45+ cells]) *100. Total cell numbers were assessed with count beads. Significance was measured with an unpaired Student *t* test. * = *P* < 0.05

**Figure 3.**
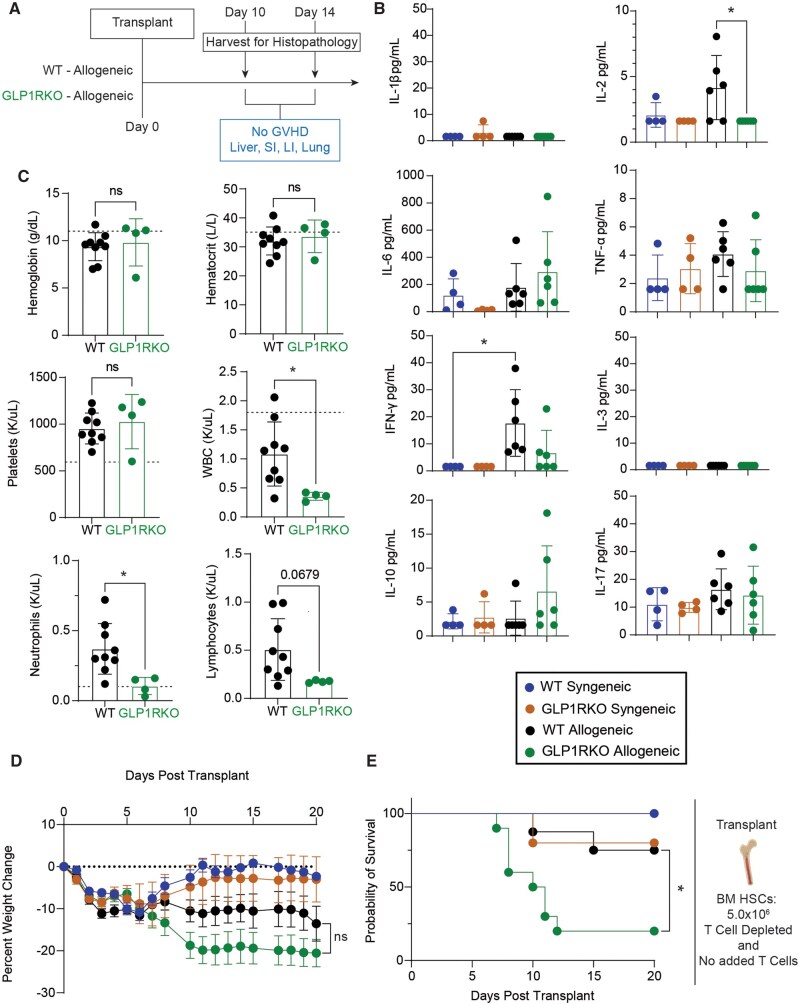
GLP1R mediated mortality in this allogeneic transplant model is not caused by GVHD. (A) Diagram of timeline sampling tissues for histopathological analysis. (SI = small intestine, duodenum) (LI = large intestine). Two separate experiments were performed for the 2 timepoints. (B) Serum concentrations of various GVHD associated cytokines measured by Luminex^®^ multiplex assay taken day 7 post-transplant (*n* = 3) syngeneic groups, (*n* = 6) allogeneic groups. Significance was assessed with 2-way ANOVA with Tukey multiple testing correction. * = *P* < 0.05. (C) CBC with differential measurements taken at day 7 post-transplant for WT (*n* = 9) and GLP1RKO (*n* = 4) allogeneic groups. Significance was measured with an unpaired Student *t* test. White blood cell (WBC) count with differential data (Neutrophils and Lymphocytes) day 7 post-transplant for WT (*n* = 9) and GLP1RKO (*n* = 4) allogeneic groups. Significance was measured with an unpaired Student *t* test. * = *P* < 0.05 (D) Percent weight change of WT and GLP1RKO mice transplanted with syngeneic or allogeneic (MHC mismatch) T cell depleted bone marrow. Percent change is calculated relative to the pre-transplant day 0 weight. Mice that die during the measurement period have their final weight included for subsequent days. Error bars indicate standard error of the mean (SEM). Significance was assessed with a 2-way repeated measures ANOVA. ns = no significant difference (*n* = 5 for both syngeneic groups, *n* = 8 for WT allogeneic, and *n* = 10 for GLP1RKO allogeneic). Figure is representative of 1 experiment. (E) Kaplan–Meier survival curve of WT and GLP1RKO mice transplanted with syngeneic or allogeneic (MHC mismatch) T cell depleted bone marrow. Significance was assessed with Mantel-Cox (Logrank) test between WT allogeneic and GLP1RKO allogeneic groups. * = *P* < 0.05 (*n* = 5 for both syngeneic groups, *n* = 8 for WT allogeneic, and *n* = 10 for GLP1RKO allogeneic). Figure is representative of 1 experiment. Created in https://BioRender.com.

All mice were conditioned for transplantation (C57BL/6J WT and GLP1RKO) with lethal irradiation through placement in a Cesium-137 irradiator. Lethal irradiation doses were split evenly between two irradiations 3 h apart to limit gastrointestinal toxicity. Mice, all on the C57BL/6J background, received 11 Gy (2 × 5.5 Gy) in a 12-chamber Mouse Pie Cage (Braintree^®^) on a rotating platform. Retro-orbital transfer of BM HSCs and T cells occurred 2 h after the last irradiation. Mice were anesthetized with a formulation of ketamine and xylazine injected intraperitoneally. Grafts were injected into the retro-orbital sinus of mice with a 1 ml TB syringe in 100 µl of serum-free RPMI. All grafts used 5.0 × 10^6^ BM HSCs. T cell containing grafts for transplant experiments in Figs. 1, 2, and 3 used 1.5 ×10^5^ T cells. For the T Reg experiment performed in Fig. S2, 5.0 ×10^6^ BALB/c BM HSCs + 1.5 ×10^5^ Balb/c T cells were given with either no T Regs, 6.0 × 10^5^ WT C57BL/6J T Regs, or 6.0 ×10^5^ GLP1RKO C57BL/6J T Regs. Mice were kept on heating pads after injections to allow proper recovery. For the duration of an experiment, mice were housed in standard caging, but cages were changed daily to ensure cleanliness and reduce risk of infection. For survival experiments, mice were marked as dead if (1) the mouse died naturally, (2) the mice lost 30% of its body weight and was euthanized, or (3) the mouse was recommended for euthanasia by animal care due to illness (having reached a humane endpoint).

### Tissue harvest for histopathology and GVHD evaluation

Mice were euthanized with pentobarbital overdose immediately prior to organ harvest. All tissues were fixed in 10% formalin. Lungs were insufflated with 10% formalin prior to excision. Livers were excised whole. Duodenum sections of the small intestine were taken (approximated as the proximal 1/3 of the distance from the pyloric sphincter to the ileocecal valve). Large intestines were cut distal to the cecum and at the rectum. Intestinal sections were cut longitudinally and fixed for 1 d in folded filter paper. Intestines were then rolled and held with a 30-guage needle and allowed to fix for an additional day. Samples were embedded in paraffin, cut, and stained with hematoxylin and eosin. Assessment of GVHD pathology was carried out by a board-certified veterinary pathologist who was blinded to the groups.

### Serum cytokine analysis

Serum was collected via cardiac puncture of recently euthanized mice. Blood was collected in snap tubes and allowed to coagulate before centrifugation. Cytokine detection was carried out via Luminex assay using x-map technology via the MagPix system. The assay was carried out by the VUMC Analytical Services Core. The MILLIPLEX MAP Mouse Cytokine/Chemokine Magnetic Bead Panel—Premixed 32 Plex—Immunology Multiplex Assay (Millipore™) was used for analysis of the following cytokines: interferon (IFN)-γ, interleukin (IL)-1β, IL-2, IL-3, IL-6, IL-10, IL-17, and tumor necrosis factor (TNF)-α.

### Flow cytometry

Flow cytometry was performed on cells from the bone marrow and peripheral blood. Isolation of bone marrow cells was identical to the method described in the “Isolation of Bone marrow-derived HSCs” section. Blood was collected via cardiac puncture of recently euthanized mice. Blood was deposited into 0.5 ml EDTA lined purple top vacuum tubes. RBC lysis was carried out, and single cell suspensions were counted for staining. Cells were first stained in a Live/Dead™ Fixable Aqua Dead Cell stain kit to assess viability. Cells were then blocked with BD Fc Block™ for 10 min in FACS buffer (3% FBS in 1× phosphate buffered saline [PBS]). Fluorescent antibodies for surface staining were added for 20 min. Cells were washed and resuspended in the eBiosciences™ FoxP3/Transcription Factor Staining Buffer set overnight at 4 °C. Intracellular staining was carried out for 45 min in manufacturer provided permeabilization buffer. Flow cytometry was performed on a 4-laser Cytek^®^ Aurora. Known quantities of 123count eBeads™ were added to samples to assess total cell quantities accurately. Analysis of flow cytometry data was performed on FlowJo software (10.8.1).

### Antibody-mediated CD90.2+ cell depletion

Mice were injected intraperitoneally with 500 μg of anti-mouse Thy1.2 (CD90.2) (30H12) depleting antibody resuspended in 100 μl of sterile PBS. Injections occurred 4 d before irradiation and transplant.

### T reg polarization

Naïve CD4^+^ T Cells were isolated from spleens of WT and GLP1RKO mice on the C57BL/6J background with the Easy Sep™ Naïve CD4+ T Cell Isolation Kit (STEMCELL™). Cells were plated on a Falcon^®^ Non-Tissue Culture Treated 24-well plates previously coated with Ultra-LEAF α-CD3ε (BioLegend^®^) at a density of 500,000 cells per well with 10 μg/ml of recombinant TGF-β (Peprotech) and 100 IU/ml of recombinant human IL-2 (NIH). Cells were cultured for 4 d before harvest for Flow Cytometry analysis and adoptive transfer with BM HSCs + splenic T cell grafts.

### Statistical analysis

All statistical analysis was carried out in GraphPad Prism 10 software. The significance of differences in survival represented in Kaplan-Meier curves were assessed with a Mantel-Cox test (Logrank). Significance for weight curves was assessed with a 2-way repeated measures analysis of variance (ANOVA). Tukey’s multiple comparisons test was used for comparison between groups. Cytokine concentration and GVHD score data were assessed with a 2-way ANOVA with Tukey’s multiple comparisons test. Significance in flow cytometry data was assessed with an un-paired *t*-test.

## Results

### GLP1R deficiency in recipients causes increased weight loss and mortality in MHC-mismatched HSCT

To assess the role of GLP1R signaling in mediating GF, we performed a set of HSCTs into WT and GLP1RKO mice on the C57BL/6J background. These mice received HSCs from C57BL/6J (Syngeneic), C3.SW-H2^b^/SnJ congenic mice (allogeneic, minor antigen-mismatch) or BALB/c (allogeneic, MHC mismatch). For each transplant, a small dose of splenic T cells from the HSC donors was included with the graft to mimic the inclusion of donor T cells in human HSCT procedures. A T cell dose of 1.5 ×10^5^ was low enough to not cause GVHD and to not overcome the residual T cells and NK cells in the host that might reject the HSC graft. Additionally, we performed the HSCTs within 2 h after the last irradiation session of conditioning, thereby decreasing the likelihood of complete host T cell and NK cell clearance upon contact with the graft. After transplant, mice were monitored for weight loss and survival (Fig. 1A). In this model, WT and GLP1RKO mice receiving syngeneic transplants fully recovered their weight after HSCT and experienced no mortality out to day 25. There was no significant difference in either weight loss (Fig. 1B) or survival (Fig. 1C). An initial dip in weight loss was observed in all groups, which is likely attributed to illness related to the Cs^137^ irradiation. Similarly, there was no significant difference in weight loss (Fig. 1D) or survival (Fig. 1E) between WT and GLP1RKO mice receiving minor antigen-mismatched grafts. Both groups recovered their weight after 60 d, and there was no significant mortality. In contrast, GLP1RKO mice experienced significantly increased weight loss (Fig. 1F) and mortality (Fig. 1G) compared to WT mice after MHC-mismatched HSCT. While 80% of WT mice survived out to 28 d, all GLP1RKO mice died within 24 d post-transplant.

### GLP1R deficiency in recipients causes graft failure (GF) of MHC-mismatched HSCs

Given the survival difference that existed between WT and GLP1RKO mice, we hypothesized that GLP1RKO mice exhibited worsened GF after MHC-mismatched HSCT compared to WT controls. To test this hypothesis, we repeated our transplant model from Fig. 1 but harvested all mice at day 7 post-transplant to assess engraftment in both the peripheral blood and bone marrow compartments. For WT and GLP1RKO mice receiving syngeneic HSCT, we compared engraftment by enumerating live CD45^+^ positive cells in the peripheral blood and bone marrow. Due to the strains available, we were unable to differentiate between recipient and donor cells for syngeneic and minor antigen-mismatch transplants. Instead, total CD45+ cell counts were used to assess graft survival. Given that mice receiving syngeneic and minor antigen-mismatched grants survived in our model, we hypothesized that we would not see a decrease in engraftment in these two conditions. As shown in Fig. 2A, there was no significant difference in engraftment between WT and GLP1RKO mice receiving syngeneic transplants. Similarly, WT and GLP1RKO mice receiving minor antigen-mismatched HSC grafts showed no difference in engraftment reflected in the peripheral blood, but to our surprise, GLP1RKO mice had more CD45^+^ cells in the bone marrow compared to WT (Fig. 2B). We were able to differentiate between donor and recipient cells for mice receiving MHC-mismatched HSC grafts, and so we assessed donor chimerism and total live donor CD45^+^ cells in WT and GLP1RKO mice receiving allogeneic HSCT from BALB/c mice (Fig. 2C). GLP1RKO mice had significantly decreased donor chimerism and total numbers of donor CD45^+^ cells in the peripheral blood and bone marrow compared to WT controls (Fig. 2D). These results validated our hypothesis that GLP1RKO mice dying in the setting of MHC-mismatched allogeneic transplant were doing so because of GF.

### GLP1R mediated mortality in this allogeneic HSCT model is not caused by GVHD

Our HSCT model included a low dose of donor T cells, and so we aimed to confirm that GVHD was not contributing to the deaths of these mice. We investigated GVHD by repeating our allogeneic HSCT model and harvested tissues on day 10 and 14 post-transplant, examining liver, lung, small intestine, and large intestine (Fig. 3A). We selected these time points because GLP1RKO mice had begun to die by day 7, and so an underlying pathology hypothetically responsible for this death would be observable at day 10 and day 14. A board-certified veterinary pathologist blinded to the groups evaluated all tissues and found no histologic evidence of GVHD in any tissue for any group. These negative histologic findings for the GLP1RKO mice at timepoints when they were dying supported our hypothesis that GVHD was not contributing to systemic illness and mortality in this model. To obtain further evidence against the presence of GVHD, we obtained serum samples at day 7 and evaluated concentrations of a host of cytokines that are typically altered in the setting of GVHD. In line with our histology findings, GLP1RKO allogeneic mice did not show an increase in IFN-γ, IL-1β, IL-2, IL-6, or TNF-α compared to WT allogeneic mice (Fig. 3B). To rule out morbidity and mortality from other hematologic complications, we performed complete blood counts (CBCs) with differentials on peripheral blood samples from transplanted mice on day 7 (Fig. 3C). We found no significant difference in hemoglobin concentration, hematocrit, or platelet counts. However, GLP1RKO mice had significantly fewer total white blood cells and neutrophils and a nearly significant trend towards fewer lymphocytes than WT mice. These findings of leukopenia on CBC support our assertion that GLP1RKO mice are experiencing increased GF in the setting of MHC-mismatch HSCT compared to WT mice.

To definitively rule out GVHD as the cause of increased mortality in GLP1RKO mice receiving MHC-mismatched HCST compared to WT, we repeated our transplant model without donor T cells. We ensured the absence of donor T cells in the HSC graft through α-CD90.2 antibody-dependent complement-mediated lysis as previously described,^41^ and we added no additional splenic T cells. WT and GLP1RKO mice receiving syngeneic T cell-depleted (TCD) HSC grafts appropriately recovered their weight, while WT and GLP1RKO allogeneic TCD groups did not. GLP1RKO mice receiving TCD HSCT experienced an increase in weight loss compared to WT counterparts that trended toward significance (Fig. 3D), and they had statistically significantly reduced survival compared to WT after transplant (Fig. 3E). The persistence of decreased survival in GLP1RKO mice in HSCT models without donor T cells strongly suggests that GVHD was not responsible for the differences in mortality observed in our earlier experiments.

### Depletion of recipient CD90^+^ cells prevents graft rejection of MHC-mismatched HSCs in GLP1RKO mice

**Figure 4.**
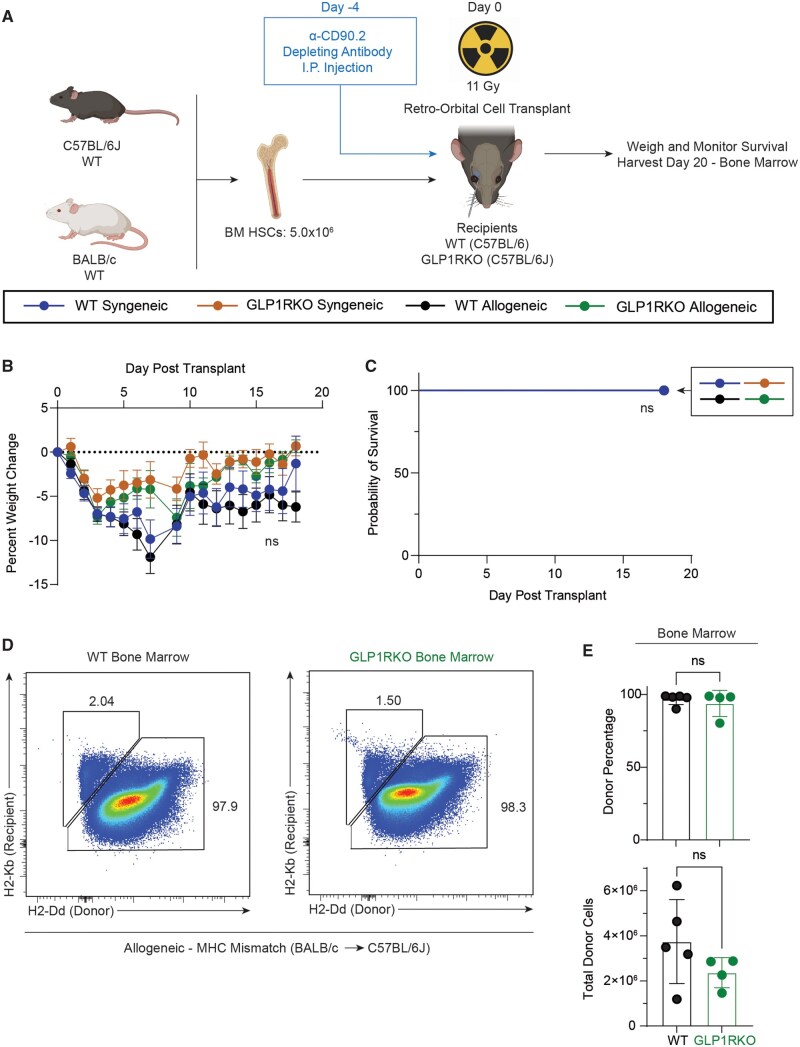
Depletion of recipient CD90^+^ cells prevents graft rejection of MHC-mismatched HSCs in GLP1RKO mice. (A) Diagram of transplant model used in Fig. 4. Anti-CD90.2 antibody injection occurred 4 d before irradiation and transplant. 11 Gy lethal irradiation was administered in 2 × 5.5 Gy doses 3 h apart. In brief, 5.0 × 10^6^ bone marrow-derived hematopoietic stem cells (BM HSCs) were transferred per graft 2 h after the last irradiation. The group legend refers to all panels in Fig. 4. (B) Percent weight change of WT syngeneic (*n* = 5), GLP1RKO syngeneic (*n* = 5), WT allogeneic (*n* = 5), GLP1RKO allogeneic (*n* = 5). Percent change is calculated relative to the pre-transplant day 0 weight. Mice that die during the measurement period have their final weight included for subsequent days. Error bars indicate SEM. Significance was assessed with a 2-way repeated measures ANOVA. ns = no significant difference. (C) Kaplan–Meier survival curve of WT syngeneic (*n* = 5), GLP1RKO syngeneic (*n* = 5), WT allogeneic (*n* = 5), GLP1RKO allogeneic (*n* = 5). ns = no significant difference. (D) Representative flow cytometry plots of bone marrow of WT and GLP1RKO allogeneic transplant mice at day 20. Cells are pre-gated on live, CD45^+^ singlet cells. “Donor” denotes the MHC haplotype originating from BALB/c, while “Recipient” denotes C57BL/6J. (E) Quantification of donor chimerism and total donor cells (BALB/c MHC+) in the bone marrow of WT (*n* = 5) and GLP1RKO (*n* = 5) allogeneic mice at day 20. Donor percentage is calculated as ([BALB/c MHC+ CD45^+^ cells]/[Total CD45^+^ cells]) *100. Total cell numbers were assessed with count beads. Significance was measured with an unpaired Student *t* test. * = *P* < 0.05. Created in https://BioRender.com.

Next, we aimed to determine if GF was mediated by recipient lymphocytes rejecting MHC-mismatched allogeneic grafts in the GLP1RKO mice. To assess this, we devised an antibody depletion model that would clear recipient mice of CD90^+^ cells, particularly T cells, before irradiation. We used an intra-peritoneal injection of α-CD90.2 antibody to effectively deplete circulating and splenic T cells within 4 d (Fig. S1A and B), and we verified that these antibodies persist long enough to deplete donor CD90.2^+^ T cells present in the HSC graft (Fig. S1C). With these recipient T cells depleted, we performed our transplants after lethal irradiation with syngeneic HSCs (C57BL/6J) and MHC-mismatched allogeneic HSCs (BALB/c) without additional T cells. Our use of a CD90.2 depleting antibody precluded the use of C57BL/6J or BALB/c (CD90.2) donors for T cells, as circulating antibody would deplete these as well (Fig. 4A). We hypothesized that CD90^+^ cell depletion in the recipient mice would abrogate the GF observed in GLP1RKO mice receiving MHC-mismatched HSC grafts. After HSCT, both WT and GLP1RKO syngeneic mice recovered weight by day 18 and did not experience mortality, aligning with our previous transplant models (Fig. 4B, -C). Both WT and GLP1RKO mice transplanted with BALB/c HSCs also recovered weight and did not experience mortality (Fig. 4B, C). This result suggested that depletion of recipient CD90^+^ cells prevented the rejection of the MHC mismatched allografts in GLP1RKO mice. We confirmed proper engraftment and a lack of rejection in these mice; no differences were observed in donor chimerism or total donor CD45^+^ cells at day 20 post-transplant in the bone marrow between WT and GLP1RKO (Fig. 4D, E).

It has been reported in the literature that GLP1R signaling influences regulatory T cell (T Reg) homeostasis.^42^^,^^43^ We hypothesized that the difference in host T cell-mediated rejection of BM HSCs in GLP1RKO mice might be due to a deficiency in T Reg suppressive function. If this hypothesis were true, supplementation of WT T Regs in a GLP1RKO recipient would more effectively reverse graft rejection than supplementation with GLP1RKO T Regs. To test this hypothesis, we polarized T Regs from naïve CD4+ splenic T cells from WT and GLP1RKO C57BL/6J mice (Fig. S2A). WT and GLP1RKO T cells achieved near identical T Reg polarization, assessed by CD25/FOXP3 double positivity on flow cytometry (Fig. S2B). Host MHC-matched WT or GLP1RKO T Regs (6.0 × 10^5^) were added to MHC-mismatched HSC + splenic T cell grafts (Fig. S2C) given to lethally irradiated GLP1RKO mice. Surprisingly, there was no benefit in prevention of weight loss or survival with the addition of either genotype of T Regs (Fig. S2D and E). This result does not support the notion that GLP1R signaling-dependent differences in T Reg function are responsible for different degrees of allogeneic HSC graft rejection.

## Discussion

Through these studies, we demonstrate that GLP1R signaling mitigates host lymphocyte-mediated HSC allograft rejection, thereby reducing the risk of GF in mice. In our models, GLP1R signaling deficiency leads to GF only following MHC-mismatched allogeneic HSCT, while syngeneic and minor antigen-mismatch transplants remain unaffected. The implication of a new genetic regulator of graft rejection is significant, as it may contribute to our understanding of the pathophysiology of this complication and lay the groundwork for further mechanistic investigation. Given the paucity of targeted therapies to prevent GF in patients receiving allogeneic HSCT, the GLP1R signaling pathway represents an exciting opportunity for study, especially in primary disorders and HSCT scenarios that associate with increased risk^4–6^ or in situations where there may be early evidence of impending graft loss.^2^ GLP1R agonists are widely used in a myriad of settings, and compounds within this class of medicinal agents with proven efficacy and safety may have substantial clinical benefit in the realm of HSCT.

The primary limitation of the study is the lack of deeper mechanistic insight. While we assert that GLP1R deficiency increases graft rejection in MHC-mismatched HSCT, more needs to be understood about how this occurs. It is possible that a lack of GLP1R signaling in the HSCT recipient indirectly increases the alloreactive potential of residual host lymphocytes through the creation of a more pro-inflammatory environment, or the GLP1R may play a more direct role on the lymphocytes themselves. Further studies are required to explore the nature of this phenomenon. Furthermore, in vivo studies assessing the efficacy of GLP1R agonists in preventing graft rejection after MHC-mismatched HSCT would be impactful in determining the therapeutic potential of these agents in this scenario. Finally, it would be informative to evaluate the effect of GLP1R on GF in variations of our model. One could assess whether the protective effect of GLP1R signaling against GF persisted with increased numbers of donor T cells or decreased intensity of the radiation-based conditioning regimen.

## Supplementary Material

vkaf251_Supplementary_Data

## Data Availability

All data that are presented in this manuscript is available upon reasonable request. Please contact the primary author at mark.rusznak.1@vanderbilt.edu or alternatively at markrusznak@gmail.com.
