# Supplementary material for: Glucagon-like peptide-1 receptor signaling deficiency exacerbates hematopoietic stem cell graft rejection in mice
Source: J Immunol. 2025 Sep 24;215(1):vkaf251. doi: 10.1093/jimmun/vkaf251 (PMC12701753; doi:10.1093/jimmun/vkaf251)
Supplement: vkaf251_Supplementary_Data [file vkaf251_supplementary_data.zip › Supplementary Figures and Legends.pdf]

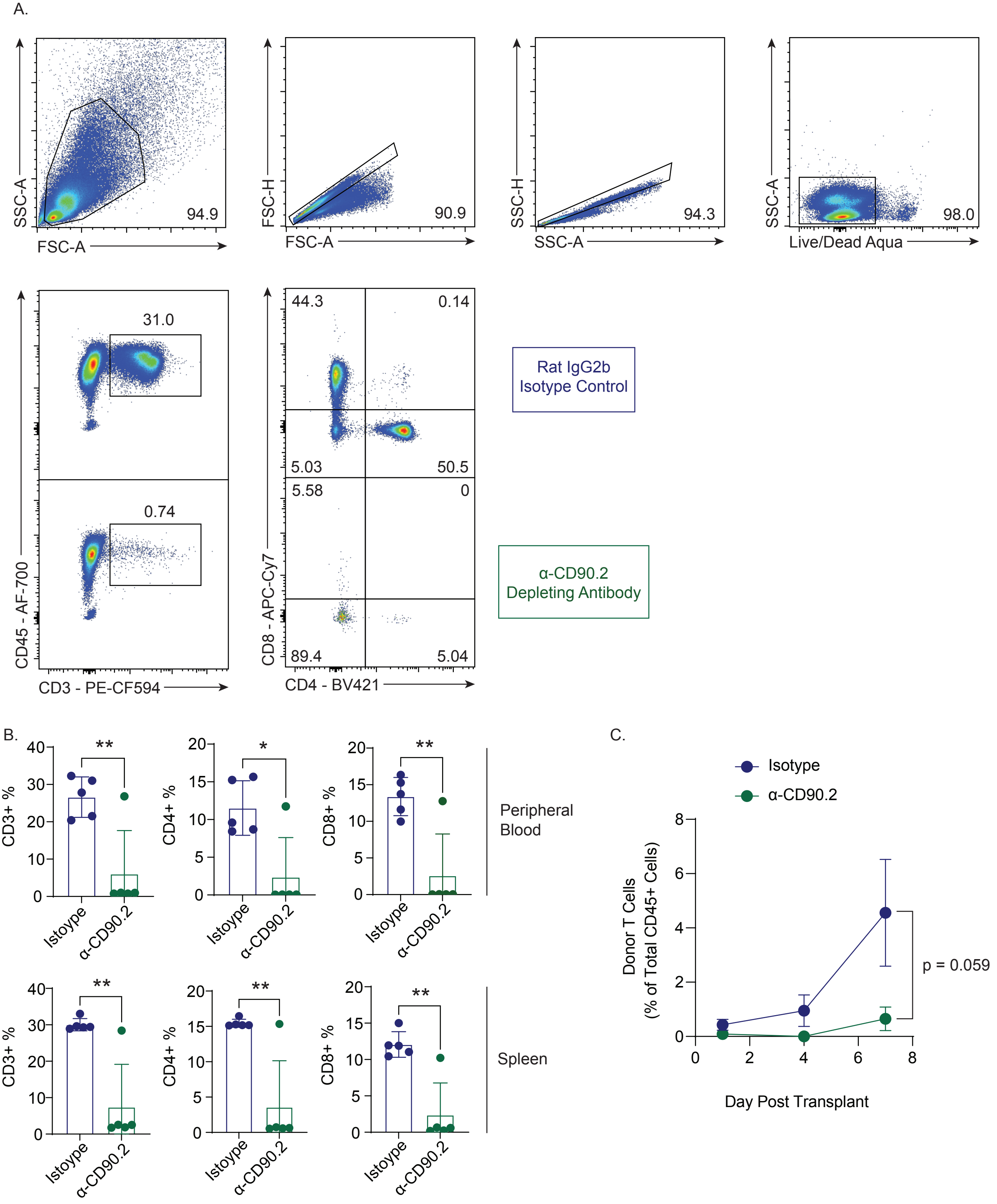
A.
SSC-A
FSC-A
94.9
FSC-H
FSC-A
90.9
SSC-H
SSC-A
94.3
SSC-A
Live/Dead Aqua
98.0
31.0
0.74
CD45 - AF-700
CD3 - PE-CF594
44.3
0.14
5.03
50.5
5.58
0
89.4
5.04
CD8 - APC-Cy7
CD4 - BV421
Rat IgG2b
Isotype Control
α-CD90.2
Depleting Antibody
B.
CD3+ %
CD4+ %
CD8+ %
Istoype
α-CD90.2
**
*
Peripheral
Blood
Spleen
C.
Isotype
α-CD90.2
Donor T Cells
(% of Total CD45+ Cells)
Day Post Transplant
p = 0.059

Supplementary Figure 1. α-CD90.2 Antibody injection successfully depletes T cells in C57BL/6J mice compared to isotype controls

(A) – Representative flow cytometry plot identifying CD4+ and CD8+ T cells in both antibody and isotype control treated mice taken from the peripheral blood.

(B) – CD3+ cells as a percentage of CD45+ cells in both isotype and antibody treated mice taken from the peripheral blood and spleen. CD4+ as a percent of all CD3+ cells and CD8+ as a percent of all CD3+ cells. (n=5) per group. Significance was assessed with an unpaired Student's t-test. *=P<0.05, **=P<0.01

(C) – Donor T cells (CD45+, CD3+, H2-Dd+, H2-Kb-) as a percentage of total CD45 cells in the peripheral blood 1, 4, and 7 days after allogeneic HSCT (MHC mismatched) into WT C57BL/6J mice treated with α-CD90.2 antibody or isotype control. Error bars indicate standard error of the mean (SEM). Significance was assessed with a two-way repeated measures ANOVA.

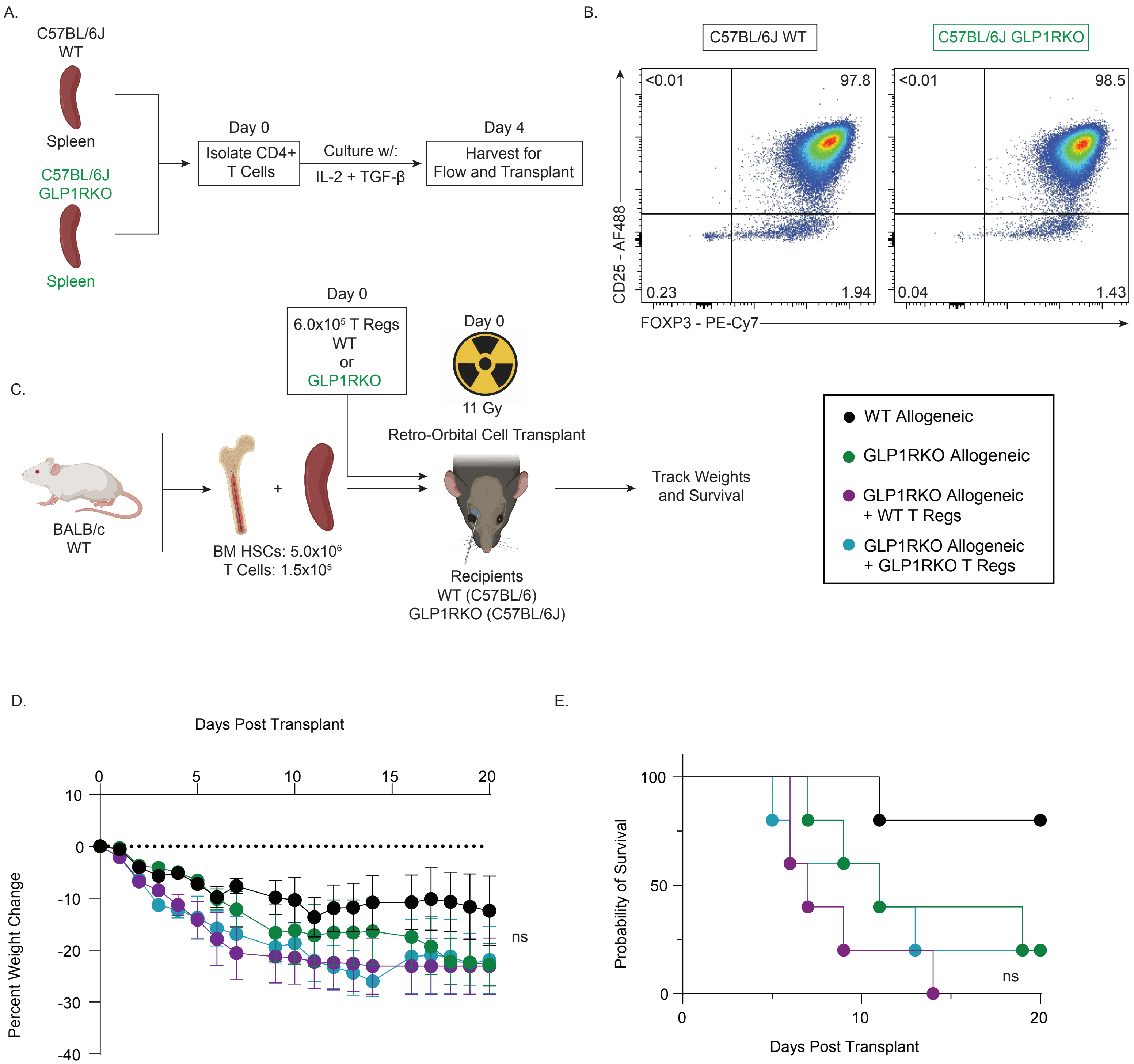
A.
C57BL/6J WT
Spleen
C57BL/6J GLP1RKO
Spleen
Day 0
Isolate CD4+ T Cells
Culture w/:
IL-2 + TGF-β
Day 4
Harvest for Flow and Transplant
B.
C57BL/6J WT
C57BL/6J GLP1RKO
<0.01
97.8
0.23
1.94
<0.01
98.5
0.04
1.43
CD25 - AF488
FOXP3 - PE-Cy7
C.
BALB/c WT
BM HSCs: 5.0x10^6
T Cells: 1.5x10^5
Day 0
6.0x10^5 T Regs WT or GLP1RKO
Day 0
11 Gy
Retro-Orbital Cell Transplant
Recipients
WT (C57BL/6)
GLP1RKO (C57BL/6J)
Track Weights and Survival
WT Allogeneic
GLP1RKO Allogeneic
GLP1RKO Allogeneic + WT T Regs
GLP1RKO Allogeneic + GLP1RKO T Regs
D.
Days Post Transplant
Percent Weight Change
ns
E.
Probability of Survival
Days Post Transplant
ns

Supplementary Figure 2. Rejection of allogeneic HSCs in GLP1RKO mice is not due to impaired T Reg function

(A) – Schematic detailing culture protocol for polarizing WT and GLP1RKO Naïve CD4+ T cells to T Regs.

(B) – Flow cytometry validation of T Reg polarization. T Regs are defined as CD3+, CD4+, FOXP3+, CD25+ cells.

(C) – Diagram of transplant model for T Reg experiments. 11 Gy lethal irradiation was administered in 2x 5.5Gy doses 3 hours apart. 5.0×10^6 bone marrow-derived hematopoietic stem cells (BM HSCs) and 150,000 splenic T cells (BALB/c) +/- WT or GLP1RKO (C57BL/6J) polarized T Regs were transferred per graft 2 hours after the last irradiation.

(D) – Percent weight change of WT and GLP1RKO mice transplanted with allogeneic (MHC mismatch) HSCs, T cells, and WT or GLP1RKO T Regs. Percent change is calculated relative to the pre-transplant Day 0 weight. Mice that die during the measurement period have their final weight included for subsequent days. Error bars indicate standard error of the mean (SEM). Significance was assessed with a two-way repeated measures ANOVA. ns = no significant difference. (n=5 for all groups). Figure is representative of one experiment.

(E) – Kaplan-Meier survival curve of WT and GLP1RKO mice transplanted with allogeneic (MHC mismatch) HSCs, T cells, and WT or GLP1RKO T Regs. Significance was assessed with Mantel-Cox (Logrank) test between WT allogeneic and GLP1RKO allogeneic groups. * =$P<0.05$ (n=5 for all groups). Figure is representative of one experiment.
